# Supplementary material for: Neuroestrogen synthesis modifies neural representations of learned song without altering vocal imitation in developing songbirds
Source: Sci Rep. 2020 Feb 27;10:3602. doi: 10.1038/s41598-020-60329-3 (PMC7046723; doi:10.1038/s41598-020-60329-3)
Supplement: Supplementary file 1 — Supplementary Information. [file 41598_2020_60329_MOESM1_ESM.pdf]

SUPPLEMENTARY INFORMATION

Neuroestrogen synthesis modifies neural representations of learned song without altering vocal imitation in developing songbirds

Daniel M. Vahaba[1-2*], Amelia Hecsh[4], Luke Remage-Healey[2-4*]

[1]Department of Biological Sciences, Program in Neuroscience, Smith College, Northampton, 01063 USA

[2]Center for Neuroendocrine Studies, [3]Neuroscience & Behavior Graduate Program, [4]Department of Psychological & Brain Sciences, University of Massachusetts Amherst, Amherst, MA 01002 USA

*Correspondence to: dvahaba@smith.edu or healey@cns.umass.edu

Supplementary Information

Figure S1 - Female songbirds temporarily prefer E2-suppressed adult song. **A**, Adult female songbirds spend more time near a speaker broadcasting adult song from systemic E2 suppressed males on day 1, but not 2, in a two-day phonotaxis experiment ($p = 0.015$). **B**, Preference ratios for FAD song relative to control song is similar across days. Additionally, we also confirmed that there was no overall side bias ($p = 0.0989$) nor inherent tendency to spend time near a specific male's song independent of treatment ($p = 0.557$). * = $p < 0.05$.

Figure S1

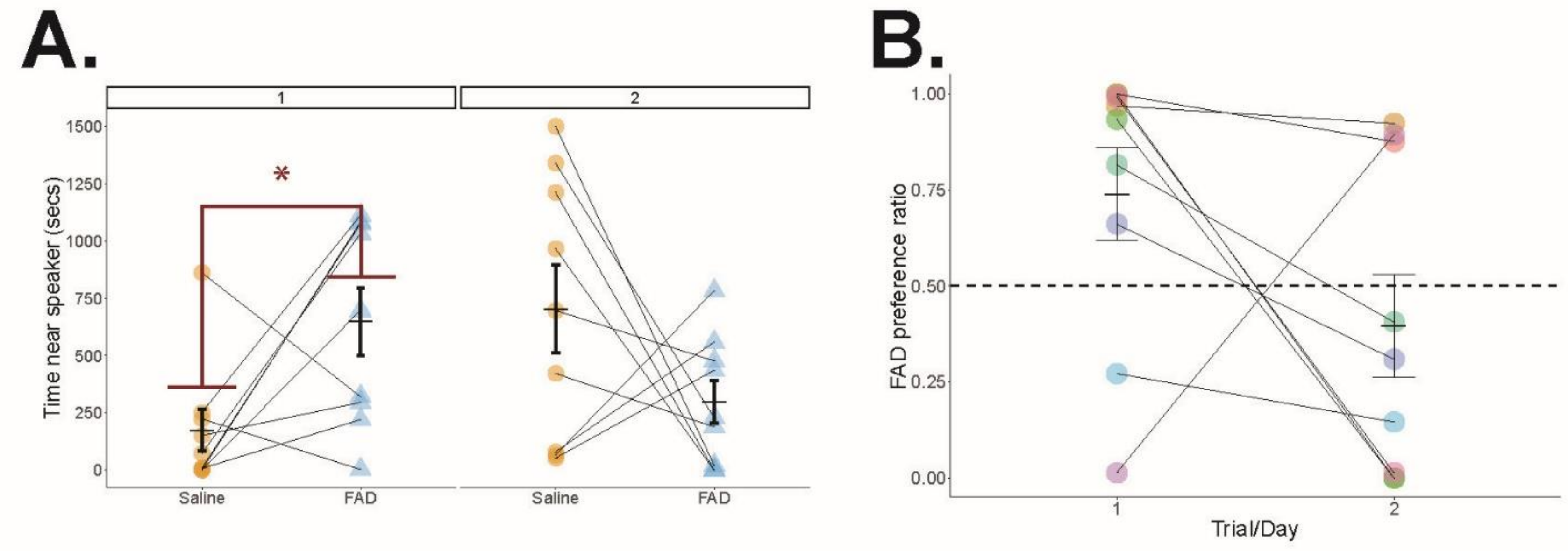
A.
1
2
Time near speaker (secs)
1500
1250
1000
750
500
250
0
*
Saline
FAD
Saline
FAD
B.
FAD preference ratio
1.00
0.75
0.50
0.25
0.00
1
2
Trial/Day

Figure S2 – Manual song similarity scoring yields similar results to automated methods. A, Example song spectrograms and their average song similarity % relative to tutor. Letters denote syllables; A’ = partial syllable derived from A. Note the seemingly high similarity of both the aCSF and FAD motif, yet divergent song similarity scores (aCSF bird = right NCM; FAD bird = right NCM; similarity score is averaged across 100 motif comparisons, see *Methods*). **B**, Manual song similarity measurements are strongly correlated with automated methods; color/shape denotes unique rater (r^2 = 0.563, p < 0.001); jitter added to reveal overlap. **C**, As with automated methods, manual song similarity scoring reveals comparable tutor song copying across treatments.

Figure S2

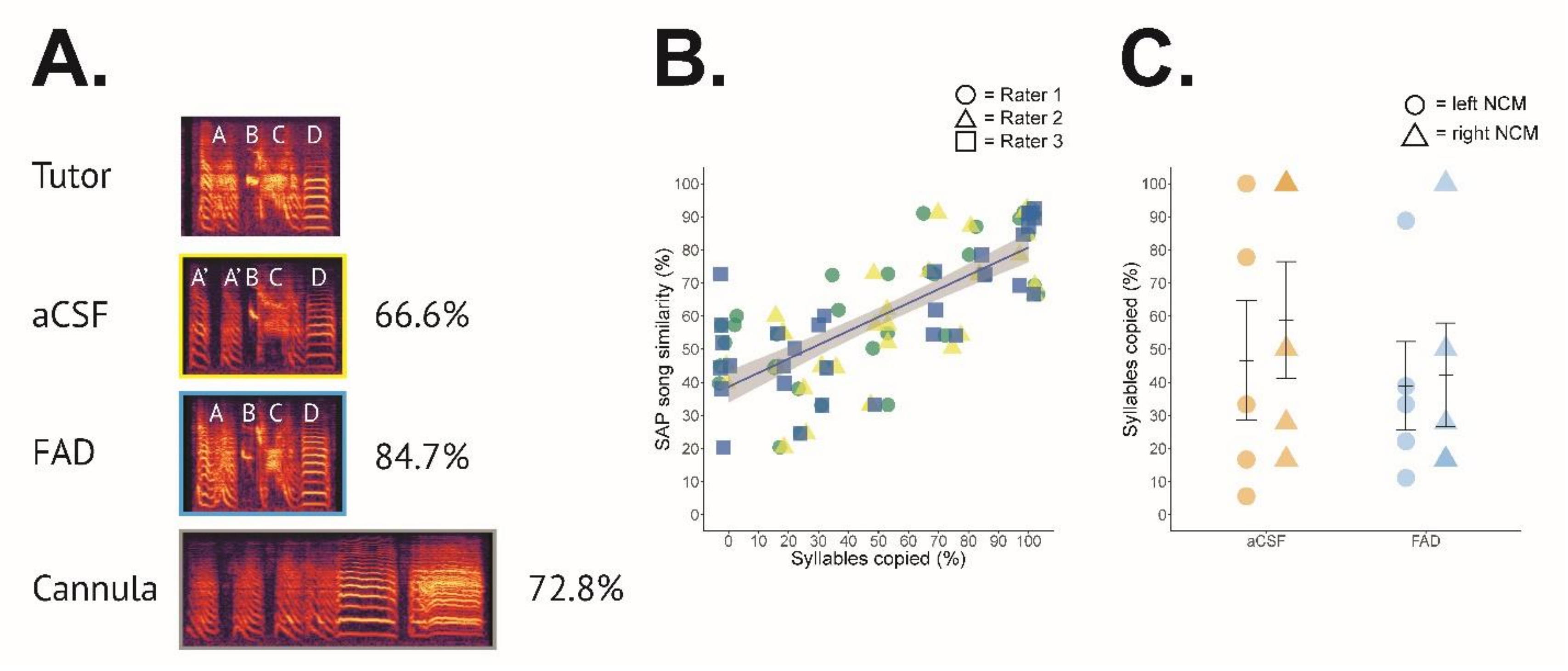
A.
Tutor
A B C D
aCSF
A' A' B C D
66.6%
FAD
A B C D
84.7%
Cannula
72.8%
B.
= Rater 1
= Rater 2
= Rater 3
SAP song similarity (%)
Syllables copied (%)
C.
= left NCM
= right NCM
Syllables copied (%)
aCSF
FAD

Figure S3 – Activity during microdialysis. **A**, Motor activity is statistically similar across treatment and tutoring days. **B**, Irrespective of treatment, microdialyzed juveniles rest less during Day 1 compared to Day 2 of tutoring. Orange = aCSF; blue = FAD; circle = left NCM; triangle = right NCM. * = $p < 0.05$

Figure S3

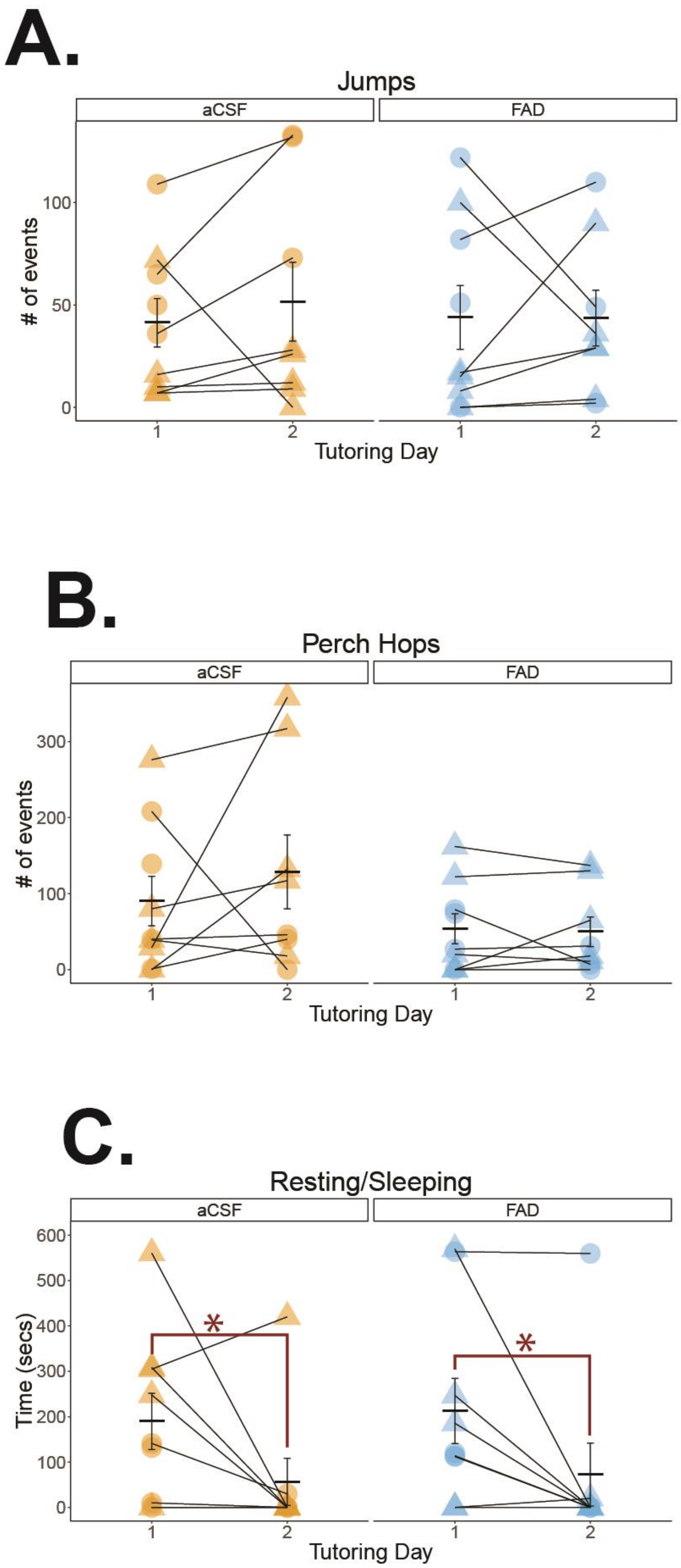

A.
Jumps
aCSF
FAD
# of events
0
50
100
1
2
1
2
Tutoring Day
B.
Perch Hops
aCSF
FAD
# of events
0
100
200
300
1
2
1
2
Tutoring Day
C.
Resting/Sleeping
aCSF
FAD
Time (secs)
0
100
200
300
400
500
600
*
*
1
2
1
2
Tutoring Day

Supp. Fig. 4 – **Song changes in formerly microdialyzed subjects after exposure to adult male song at 130 dph. A,** Spectrogram examples from one FAD-treated microdialysis subject that changed its song following exposure to adult male conspecifics at 131 dph. *Left column*: song at 130 dph; *Right column*: after the subject was exposed to conspecific adult males following isolation with an adult female conspecific (131 dph), song was recorded at 356 dph preceding electrophysiology experiments. **B,** Histogram demonstrating that treatment had no bearing on whether microdialysis subjects altered their adult song after exposure to conspecific males. All birds with aberrant song at 130 dph changed their song after exposure to other male conspecifics.

Figure S4

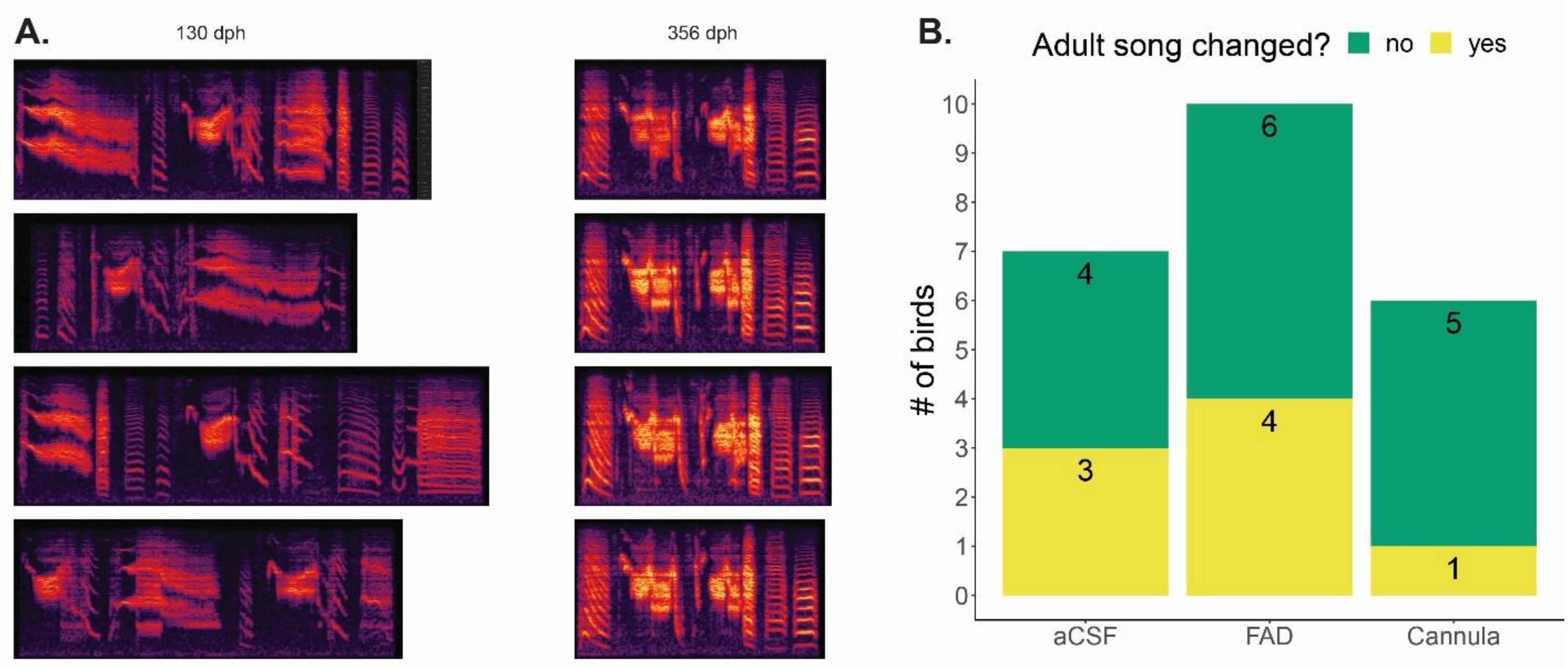
A.
130 dph
356 dph
B.
Adult song changed?
no
yes
# of birds
0
1
2
3
4
5
6
7
8
9
10
4
3
6
4
5
1
aCSF
FAD
Cannula

Figure S5 – Contralateral d' selectivity in single NCM and HVC neurons. A, NCM; statistical analyses were performed for TUT and BOS, only (see *Results*). Other stimuli plotted for visual comparison. Irrespective of stimulus, responses were significantly higher in aCSF treated subjects in both ipsilateral and contralateral NCM. **B,** HVC; statistical analyses were performed for TUT and BOS, only (see *Results*). Other stimuli plotted for visual comparison. Both TUT and BOS selectivity were statistically similar in contralateral HVC.

Figure S5

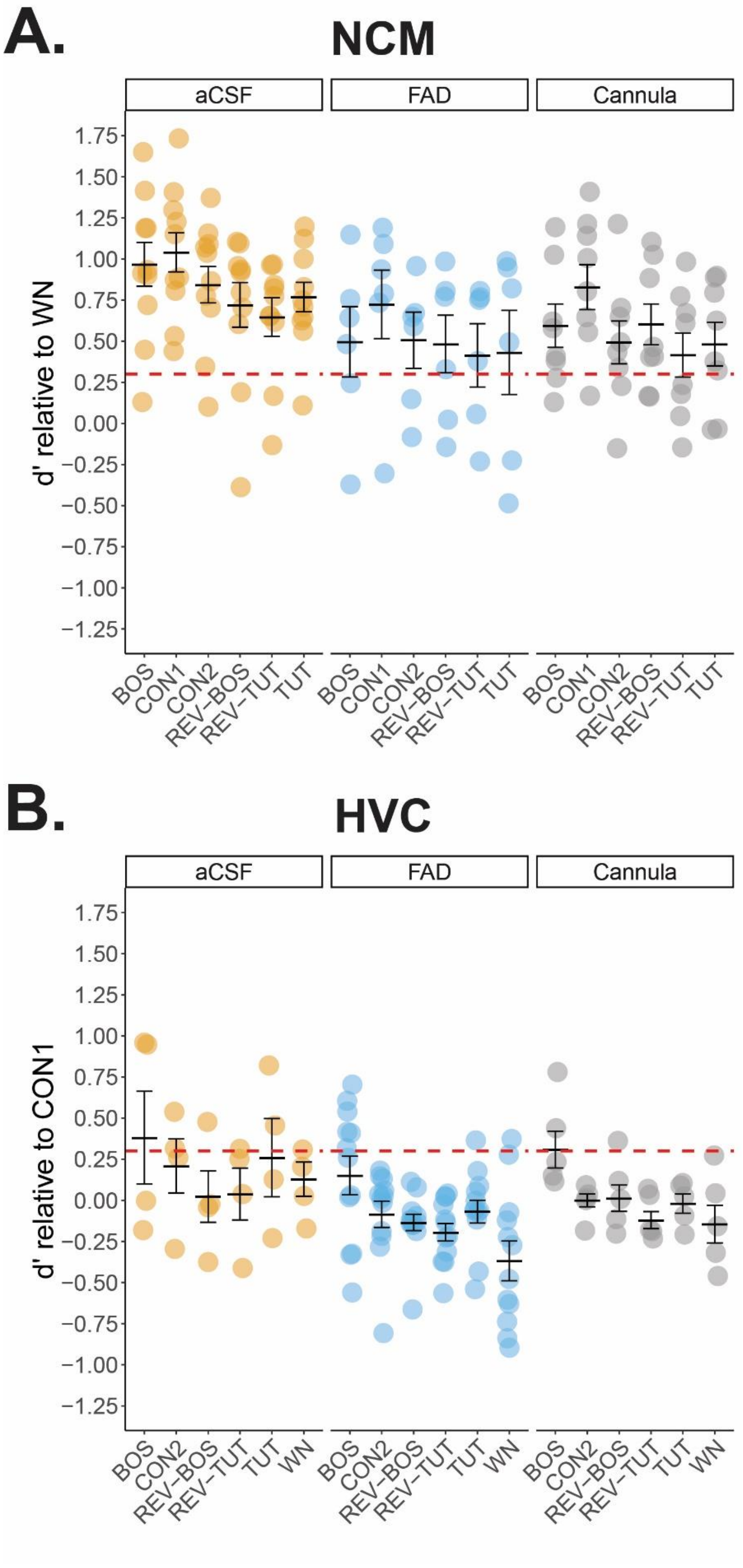
A.
NCM
aCSF
FAD
Cannula
d' relative to WN
1.75
1.50
1.25
1.00
0.75
0.50
0.25
0.00
−0.25
−0.50
−0.75
−1.00
−1.25
BOS
CON1
CON2
REV−BOS
REV−TUT
TUT
B.
HVC
d' relative to CON1
WN
